# Reviewed article: Soares LNS, Cabral AEA, Luz SBA, Lourenço MAG, Pazinatto RB, Santos BSM, Melo LA. Sociodemographic, general health and oral health factors associated with difficulty in eating among elderly people in Brazil: a cross-sectional study, 2019. Epidemiol Serv Saude. 2025:34;e20240678

**DOI:** 10.1590/S2237-96222025v34e20240678.b

**Published:** 2025-08-04

**Authors:** Reyce Koga

**Affiliations:** Universidade de São Paulo, Política, Gestão e Saúde

**Table:** 

Rating	Excellent	Good	Average	Below average	Poor
Interest	✓				
Quality	✓				
Originality		✓			
Overall	✓				

**Table:** 

Questionnaire	Yes	No	Not applicable
Does the manuscript contain new and significant information to justify publication?	✓		
Does the Abstract (Summary) clearly and accurately describe the content of the article?	✓		
Is the problem significant and concisely stated?	✓		
Are the methods described comprehensively?	✓		
Are the interpretations and conclusions justified by the results?	✓		
Is adequate reference made to other work in the field?	✓		

## Manuscript Structure

Length of article is: Adequate

Number of tables is: Adequate

Number of figures is: Adequate

Please state any conflict(s) of interest that you have in relation to the review of this paper (state “none” if this is not applicable): None

## Comments

Dear authors,

Comentários:

1.Título

Título genérico e pouco atrativo. Trazer os resultados do estudo pode torná-lo mais atrativo.

2. Resumo

A parte da descrição da análise estatística pode ser mais bem descrita. Sugiro citar que também foi feita a análise bruta antes da ajustada.

3. Introdução

Não vejo motivo para a repetição de referência no primeiro e segundos períodos do primeiro parágrafo e no segundo e terceiros períodos do segundo parágrafo, já que se trata da mesma referência.

3˚ parágrafo, linha 32 e 34. O termo “oral” se refere à fala, mudar para “higiene bucal” na linha 32 e na linha 34 para “saúde bucal”. Ajustar também na linha 33 da página 7 da discussão.

4. Métodos

Seção análise estatística: está subentendido que foram feitas razões de prevalência bruta, pois os dados são apresentados na tabela 1, porém acho importante que o texto mencione isso.

5. Resultados

Organizar as tabelas 3 e 4 com as variáveis na linha superior a referência e na linha inferior o desfecho, pode facilitar o entendimento. Em ambas as tabelas também faltam o número de participantes no título.

6. Discussão

Adequada e analisa os resultados de forma satisfatória.

7. Referências

Julgo importante citar a fonte de extração do banco de dados da PNS.

